# Identifying Barriers and Supports to Breastfeeding in the Workplace Experienced by Mothers in the New Hampshire Special Supplemental Nutrition Program for Women, Infants, and Children Utilizing the Total Worker Health Framework

**DOI:** 10.3390/ijerph16040529

**Published:** 2019-02-13

**Authors:** Eric A. Lauer, Karla Armenti, Margaret Henning, Lissa Sirois

**Affiliations:** 1 Institute on Disability, New Hampshire Occupational Health Surveillance Program, University of New Hampshire, College of Health and Human Services, Durham, NH 03824, USA; karla.armenti@unh.edu; 2 Department of Public Health, Keene State College, Keene, NH 03435, USA; mhenning@keene.edu; 3 State Director, Special Supplemental Nutrition Program for Women, Infants, and Children, New Hampshire Department of Health and Human Services, Concord, NH 03301, USA; lissa.sirois@dhhs.nh.gov

**Keywords:** total worker health, breastfeeding, industry, workplace accommodations, work environment, work culture, work policy, health promotion, occupational health surveillance

## Abstract

Variations in the barriers and contributors to breastfeeding across industries have not been well characterized for vulnerable populations such as mothers participating in the Special Supplemental Nutrition Program for Women, Infants, and Children (WIC). Our study used the Total Worker Health Framework to characterize workplace factors acting as barriers and/or contributors to breastfeeding among women participating in the New Hampshire WIC. Surveys were collected from WIC mothers (*n* = 682), which asked about employment, industry, and workplace accommodation and supports related to breastfeeding in the workplace. We found workplace policy factors supporting breastfeeding (i.e., having paid maternity leave, other maternity leave, and a breastfeeding policy) varied by industry. Women in specific service-oriented industries (i.e., accommodation and retail) reported the lowest rates of breastfeeding initiation and workplace supports for breastfeeding and pumping. Further, how a woman hoped to feed and having a private pumping space at work were significantly associated with industry, breastfeeding initiation, and breastfeeding duration. A substantial portion of women reported being not sure about their workplace environment, policies, and culture related to breastfeeding. Additional studies with larger sample sizes of women participating in WIC are needed to further characterize the barriers to breastfeeding associated with specific industries.

## 1. Introduction

Over the last two decades, the global public health community established that working outside the home was negatively associated with breastfeeding [1,2,3,4]. The World Health Organization has recently recognized the need for increased supports to improve breastfeeding duration and initiation rates, recommending women breastfeed for two years [5]. However, research in the United States (U.S.) found only 49% of women breastfeed for 6 months and breastfeeding initiation was impacted by working or planning to work postpartum [1,3,6,7]. Studies have found that breastfeeding incidence and duration were lower among employed, working-age women [1,3,8,9,10]. 

Moreover, women planning to work full-time postpartum were less likely to initiate breastfeeding than women who planned to work part-time and women were more likely to cease breastfeeding the first month prior or subsequent to returning to work [1,4]. Employment was also associated with breastfeeding less than two to three months postpartum [2]. Women who return to full-time employment six to twelve weeks postpartum were more than 50% less likely to meet their breastfeeding intentions, and women who return to full-time employment less than 6 weeks postpartum were more than twice as likely to not meet their breastfeeding intentions, compared to women who do not work [11].

Having identified this disparity, recent research has recognized the need to explore factors that influence breastfeeding cessation when returning to work [12]. Mothers themselves report multiple barriers to breastfeeding once returning to work, such as a lack of flexibility in the work schedule to allow for milk expression; lack of accommodations to express and/or store human milk; and concerns about support from supervisors and colleagues [13,14]. A woman’s breastfeeding duration is also influenced by the existence and quality of maternity leave including its length, paid or unpaid status, and the attitudes, policies, and practices at her place of employment [15]. Among working women or women returning to work, research has found breastfeeding initiation and duration were lower for low-income women and women with less than a high school education [4,11]. However, this literature does not characterize specific, practical worksite factors that influence breastfeeding disparities among vulnerable populations.

### 1.1. Special Supplemental Nutrition Program for Women, Infants, and Children

Breastfeeding disparities experienced by low-income women due to individual, social, and environmental barriers are well documented [5,16]. Breastfeeding prevalence among low-income women, specifically women enrolled in the Special Supplemental Nutrition Program for Women, Infants, and Children (WIC), continues to be below national targets established in Healthy People 2020 [17]. Nationally, WIC mothers have lower rates of breastfeeding than non-participants, despite WIC’s efforts to encourage breastfeeding through the Loving Support Makes Breastfeeding Work campaign and the WIC Peer Counseling Program [18,19,20]. Among WIC participants, barriers to breastfeeding include embarrassment toward breastfeeding in public, early return to work or school, infant behavior, lactation complications, lack of self-efficacy, low income, limited social support, less education, and unsupportive childcare [21,22,23,24].

The type of employment generally obtained by WIC eligible mothers further contributes to the disparities found in the U.S. WIC eligible mothers are more likely to have low-income jobs in childcare, home healthcare, or in one of the service industries [25]. These jobs are less likely to have flexible schedules or have paid-for breaks to express breast milk, both of which contribute to the mothers decision-making about continuing to breastfeed or early weaning [16]. These industries also usually lack workplace lactation policies and supports, which influence a woman’s choice to continue breastfeeding upon returning to work [12].

The studies among WIC mothers identify a vulnerable population at risk of experiencing disparities in breastfeeding, which vary by employment type. However, employment supports and policies for breastfeeding are geographically and industry-dependent. For example, in New Hampshire (NH), according to the 2016 NH WIC Pediatric Nutrition Surveillance data, 76.8% of WIC mothers initiate breastfeeding after delivery, compared to 87.4% of all mothers in New Hampshire [26,27]. Further, previous studies have found that NH WIC mothers are less likely to have ever breastfed than other mothers in the U.S. or NH, and WIC mothers’ breastfeeding duration was significantly related to employment status [21,28]. Additional state-specific research is needed to better understand and characterize breastfeeding disparities across employment types among WIC mothers.

### 1.2. The Total Worker Health Program and Benefits of Breastfeeding

The Total Worker Health (TWH) Program, created by the National Institute for Occupational Safety and Health (NIOSH) in 2012, supports protective and preventive efforts to improve the health and well-being of workers [29]. The TWH framework for worker well-being provides agreed-upon definitions and measures of worker well-being based on comprehensive, multidisciplinary literature reviews and expert panels consisting of occupational safety and health researchers [30]. The framework provides an opportunity to explore and characterize worksite factors that could improve breastfeeding behavior and supports for WIC mothers.

Breastfeeding-friendly worksites have been associated with benefits for both mothers and employers that include reduced employee absenteeism, increased employee retention, increased employee morale and loyalty, healthcare cost savings, and positive public relations and company image [31,32]. Although having space alone is not associated with increased duration of breastfeeding, women need the knowledge, support, encouragement, and environment to be successful in reaching their goals [33]. For every 1000 babies not breastfed, there are an extra 2033 physician visits, 212 days of hospitalization, and 609 prescriptions written [34]. 

### 1.3. Study Considerations

To the best of our knowledge there have been no studies exploring breastfeeding behavior across industries among WIC mothers. Using the TWH framework to characterize workplace factors for breastfeeding, this study examined the barriers and contributors to breastfeeding experienced by mothers in the NH WIC Program and focused on identifying workplace polices, supports, and practices that encourage or discourage breastfeeding after returning to work.

## 2. Methods

### 2.1. Study Design and Site Selection

This study used a cross-sectional design to survey women utilizing the WIC services in NH. In order to represent the entire state, all four local agencies providing WIC services in NH with catchment areas covering all ten counties in the state were utilized in the study.

### 2.2. Survey Development

As a first step towards designing this survey, a state-wide collaboration of multiple NH agencies, local coalitions, programs, and universities partnered to inform a questionnaire of breastfeeding in the workplace among NH WIC program participants. The team consisted of faculty and staff from the Institute on Disability, New Hampshire Occupational Health Surveillance Program in the University of New Hampshire’s College of Health and Human Services, Keene State College Department of Public Health and Department of Health Science, New Hampshire Department of Health and Human Services’ Division of Public Health Services, the Director of the New Hampshire WIC Nutrition Program, and a subcommittee of the New Hampshire Breastfeeding Taskforce. Based on a literature review of breastfeeding in the workplace and using focus groups, online feedback, and expert commentaries and reviews, a nine-page survey was iteratively developed to identify and measure person- and environment-level barriers and supports to breastfeeding initiation and continuation in the workplace.

Informed by prior breastfeeding studies of women using NH WIC services, survey questions were categorized into worker well-being domains operationalized by the NIOSH TWH Program [29]. The domains included in this study were (1) workplace physical environment and safety climate, (2) workplace policies and culture, (3) health status, and (4) work evaluation and experience [30]. The survey items used in this study identified concepts in the workplace related to the supports, knowledge/training, policies, physical space, and culture that encourage or discourage breastfeeding after returning to work. 

Question content (i.e., concepts, topics, and phrasing) was based broadly on the existing literature and the state-wide partnership formed for this study. Survey questions were also included from (1) the Monadnock Region’s Community Coalition for the Promotion of Breastfeeding Survey implemented in the Keene and Manchester NH WIC Programs, and (2) lactation support questions from the Centers for Disease Control and Prevention’s Worksite Health ScoreCard [21,35]. Where possible, questions offered multiple choice responses similar to the‘Monadnock Region’s Promotion of Breastfeeding Survey, where the respondent could check more than one response [21]. This research, combined with the NH Breastfeeding Taskforce’s experience and feedback, found breastfeeding to be a sensitive topic and recommended an anonymous survey with multiple choice responses to allow mothers to answer more honestly and in a fast, efficient manner.

When answering work questions, survey participants were instructed to reply for their current job, and if they were currently on maternity leave or recently left their job, they were asked to respond for their most recent job. Further, some survey items also included a step-wise, conditional design where participants were only asked to answer some secondary questions that were dependent on responses to the previous question in the survey. Topics with conditional questions included maternity leave, breastfeeding policies, break times for pumping at work, reactions to breastfeeding at work, and supports for breastfeeding longer at work.

The paper survey was pilot-tested for feasibility in the spring of 2016 using a random 1% of each the four local WIC agencies caseload. The results of the pilot informed the survey design and ensured the viability of a multi-agency study design. Once the survey was finalized, the University of New Hampshire Institutional Review Board granted approval for data collection. The study was conducted in accordance with the Declaration of Helsinki and the protocol was approved by the University of New Hampshire Research Integrity Services Institutional Review Board as IRB#6491.

### 2.3. Survey Eligibility and Administration.

Study eligibility criteria required women be enrolled in the WIC program and the birth mother of their child or that their child was enrolled in the WIC Program. Each local WIC office’s breastfeeding coordinator directed survey implementation at their respective agency. WIC participants were recruited by referrals from WIC staff, and flyers in the WIC offices. Surveys were offered to all women participating in the NH WIC program that visited local agency offices during August, September, and October of 2016. Surveys were administered in English and staff were available for respondent questions while completing the survey. Immediately prior to administration, respondents received instructions as to the purpose, audience, risks, benefits, and completion of the survey. Surveys were completed immediately onsite and took approximately 15 minutes to finish.

### 2.4. Sample Size

A total of 682 mothers responded to the survey. This degree of survey participation represents approximately 5.0% of the total WIC caseload in NH at the time of the study.

### 2.5. Data Collection

Paper surveys were completed during an office visit to WIC agencies while participants were at a WIC appointment receiving benefits or waiting to be called for their appointment. As the study proceeded, agency staff collected and periodically sent surveys to the research team. A student entered each respondent’s data into an online version of the survey designed in Qualtrics Software to create an excel data file.

### 2.6. Variables

#### 2.6.1. Demographics

Age was coded as 15 to 17, 18 to 34, and 35 and over. Race/ethnicity was coded as non-Hispanic White, and Other.

#### 2.6.2. Breastfeeding

“Breastfeeding initiation” was based on three questions including: (1) declared breastfeeding status (currently breastfeeding, previously breastfed, planning to breastfeed, or never breastfed); (2) “One week after you gave birth, how were you feeding your baby?”, with responses of breast milk only, formula only, both breast milk and formula; and (3) “How old was your baby when you stopped breastfeeding completely?”, with responses of one through thirteen months or baby has not stopped breastfeeding. Ever having breastfed was defined as reporting currently breastfed, previously breastfed, feeding breast milk one week after birth (only or in combination with formula), having stopped breastfeeding, or having a baby that has not stopped breastfeeding. Never having breastfed was defined as reporting planning to breastfeed, never having breastfed, not feeding with breast milk the first week after birth, not having stopped breastfeeding, and not having a baby that continues to breastfeed. 

“Duration of breastfeeding” was based on the declared breastfeeding status and the question: “How old was your baby when you stopped breastfeeding completely?” (see “Breastfeeding initiation” for all response categories). Breastfeeding for less than 4 months was defined conditionally on having reported previously breastfeeding and having stopped breastfeeding after one, two, or three months. Breastfeeding for greater than or equal to 4 months was defined conditionally on having reported previously breastfeeding and having stopped breastfeeding after four or months. 

“Reason for stopping breastfeeding” was based on the question: “What were your reasons for stopping breastfeeding?” Responses were categorized as physiological (difficulty nursing or latching; not enough milk, milk dried up; too painful, too hard, nipples too sore; I got sick, my baby got sick, had to stop for medical reasons), other commitments (too much time, I went back to school, I went back to work), and met goal (I met my goal). More than one reason could be reported.

#### 2.6.3. Employment and Industry

“Employment status” was based on the question: “What is your employment status?” Responses were categorized as full-time, part-time, and other. Other included individuals not in the labor force, unemployed, in school, disabled, seasonal workers, and/or stay at home mothers. 

“Industry” was coded according to 2012 Census Industry Classification Codes. Industry responses were combined into broader categories to increase the number of responses in those categories for analysis purposes. “Accommodation, food, and hospitality” included responses from women working in restaurant, travel and hotel jobs. “Healthcare” included women working in healthcare or in a hospital setting as well as working in home healthcare, as a licensed nursing assistant, or in an assisted living or nursing home environment. “Retail” included women working in grocery, clothing, convenience, and department stores. “Other” included all other industries with sample sizes too small to consider separately. This included but was not limited to education, social assistance services, manufacturing, and other services. Study participants were asked to respond to the industry question only if they were currently employed or planning to return to work.

#### 2.6.4. During Pregnancy

“Hoping to feed” was based on the question: “While you were pregnant, how had you hoped to feed your baby?”, with responses of breastfeeding only, formula only, or a combination of breastfeeding and formula. “Received information” was based on the question: “Did you receive information about breastfeeding during pregnancy?”, with responses of yes or no.

#### 2.6.5. Total Worker Health Well-Being Domains

##### Policies and Culture

“Paid maternity leave” was based on the question: “Does your workplace offer paid maternity leave and is it separate from any other leave such as sick and or vacation leave?”, with responses of yes, no, and “not sure.” Conditional on having reported no to having paid maternity leave, “other maternity leave” was based on the question: “can employees take paid maternity leave using other leave such as sick time or vacation time?”, with responses of yes, no, and ”not sure.” “Breastfeeding policy” was based on the question: “Does your workplace have a written policy on breastfeeding or pumping?”, with responses of yes, no, and ”not sure.” “Seen policy” was conditional on having reported yes to having a breastfeeding policy at work and based on the question: “Have you seen or do you have a copy of the written policy on breastfeeding at your workplace?”, with responses of yes, no, and ”not sure.” “Pumping break times” was based on the question: “Does your workplace provide break times to allow mothers to pump breastmilk?”, with responses of yes, no, and ”not sure.” Conditional on having reported yes to having break times for pumping, “flexible breaks” was based on the question: “Is the break time flexible (e.g., you can take it when you need to)?”, with responses of yes, no, and ”not sure.”

##### Physical environment and Safety Climate

“Private pumping space” was based on the question: “Does your workplace have a private space (NOT bathroom, or closet) for you to use a breast pump?”, with responses of yes, no, and ”not sure.” “Onsite items” was based on the question: “Does your workplace offer the following items on-site for employees to use when expressing breastmilk?”, with responses categorized as utilities (electrical outlet and/or nearby sink) and physical (chair and/or space with locked door). More than one item could be reported. “Supportive coworkers” was based on the question: “Are your co-workers supportive of mothers who need to use a breast pump during work?”, with responses of yes, no, and ”not sure.” “Supportive supervisor” was based on the question: “Is your supervisor supportive of mothers who need to use a breast pump during work?”, with responses of yes, no, and ”not sure.”

##### Health Behavior

“Pumped at work” was based on the question: “Have you had to use a breast pump at work?”, with responses of yes, no, and “not sure.”

##### Work Evaluation and Experience

“Reaction to pumping” was conditional on having reported yes to having pumped at work and based on the question: “What type of reaction, if any, have you received from those you work with?”, with responses categorized as negative (negative reactions only or some positive and some negative reactions) and positive (no reactions given or positive reactions only). “Pumped longer if easier” was based on the question: “Would you have continued breastfeeding longer if it was easier to pump at work?”, with responses of yes, no, and ”not sure.” Conditional on having reported yes to breastfeeding longer if it was easier at work, “factors” asked people to report what factors would have made it easier, with responses categorized as policy (a copy of the company policy, supportive coworkers, supportive supervisor, or flexible time/hours), and environment (a place to store breastmilk or a private space for pumping). More than one factor could be reported.

### 2.7. Statistical Analysis

All analyses were conducted using SAS Version 9.4 (SAS Institute Inc., Cary, NC, USA). Univariate and bivariate methods were used to estimate counts and percentages. Bivariate associations in contingency tables were tested either using Fisher’s exact tests or Fisher’s exact tests with Freeman and Halton’s adaptations for non-standard RowxColumn tables [36]. Monte Carlo estimation with 10,000,000 samples was used to calculate Fisher–Freeman–Halton statistics for tables within industry. Statistical significance was determined based on an alpha of 0.05 with Bonferroni corrections dependent on the number of questions in a given worker well-being domain or survey topic. Estimates were considered marginally significant if they met the alpha criteria of 0.05 but were no longer significant after Bonferroni correction. Analyses and categorization of questions about demographics, employment status, and during pregnancy included not reported as a category to account for nonresponse. For Total Worker Health well-being questions, directed towards people who were employed, on maternity leave, or recently employed, nonresponse was not included.

## 3. Results

### 3.1. Breastfeeding Initiation

Table 1 presents the maternal demographic, employment, and pregnancy characteristics for the overall sample and stratified by never or ever having initiated breastfeeding. The majority of our sample was between the ages of 18 to 34, non-Hispanic White, and approximately 50% were employed full- or part-time. Greater percentages of women aged 35 and over had ever breastfed, compared to women who had never breastfed (13.5% vs. 8.4%, *p* = 0.025). There were no differences in race/ethnicity or employment status by breastfeeding initiation (*p* = 0.123 and *p* = 0.723, respectively). By industry, a greater percentage of women who had ever breastfed worked in healthcare (26.3% vs. 20.0%) and a smaller percentage worked in accommodation and retail (20.4% vs. 33.8% and 16.1% vs. 24.6%) than women who had never breastfed (*p* = 0.011). Among women who had breastfed, 64.2% had hoped to exclusively breastfeed during pregnancy, compared to 12.6% of women who had never breastfed (*p* = 0.000). Among women who had never breastfed, approximately 40.3% had hoped to breastfeed during pregnancy. There was no difference in the percentage of women who had received information about breastfeeding during pregnancy by breastfeeding initiation status (*p* = 0.224).

Table 2 presents maternal workplace characteristics for the overall sample and stratified by never or ever having initiated breastfeeding. Within the policies and culture domain, a greater percentage of women who had ever breastfed had break times for pumping (49.7% vs. 25.9%, *p* = 0.000), compared to women who had never breastfed. There was no difference in having paid maternity leave, other maternity leave, a breastfeeding policy, having seen the workplace breastfeeding policy, or having flexible break times for pumping by breastfeeding initiation status (all *p* > 0.400). Within the physical environment and safety climate domain, by breastfeeding initiation status (ever vs. never), there were significant differences in having private spaces for pumping (40.8% vs. 19.0%, *p* = 0.000), supportive coworkers (51.6% vs. 36.6%, *p* = 0.014), and supportive supervisors (51.0% vs. 38.0%, *p* = 0.011).

In addition, in Table 2, the percentage of women reporting “not sure” was notable for all workplace factors. Women who never breastfed consistently had greater percentages of “not sure” than women who had ever breastfed. Across significant associations for having break times for pumping, private pumping space, supportive coworkers, and supportive supervisors, the percentages of women reporting “not sure” was greater for women who had never breastfed (range: 41.8–59.2%) than women who had ever breastfed (range: 23.1–40.4%).

### 3.2. Duration of Breastfeeding

Table 3 presents the maternal demographic, employment, pregnancy, and breastfeeding characteristics stratified by duration of breastfeeding (less than 4 months or 4 months or longer). There was no difference in age, race/ethnicity, employment status, or industry by duration of breastfeeding (all *p* > 0.099). During pregnancy, 73.8% of women who breastfed for 4 months or longer hoped to only breastfeed, compared to 50.4% of women who breastfed less than 4 months (*p* = 0.000), and there was no difference in the percentage of women who receive information about breastfeeding during pregnancy by duration of breastfeeding (*p* = 0.736). Across reasons for stopping breastfeeding, compared to women who breastfed 4 months or longer, a greater percentage of women who breastfed for less than 4 months stopped breastfeeding for physiological reasons (86.6% vs. 64.7%, *p* = 0.000), a smaller percentage stopped breastfeeding when they met their breastfeeding goal (1.5% vs. 28.1%, *p* = 0.000), and there was no difference in the percentage of women who stopped breastfeeding due to other commitments (*p* = 0.888).

Table 4 presents the maternal workplace characteristics stratified by duration of breastfeeding (less than 4 months or 4 months or longer). Within the policies and culture domain, there was one marginally significant difference by duration of breastfeeding. A greater percentage of women who breastfed 4 months or longer had break times for pumping, compared to women who breastfed less than 4 months (53.3% vs. 39.8%, *p* = 0.032, not significant after Bonferroni correction). Within the physical environment and safety climate domain, there were two marginally significant differences by duration of breastfeeding. Compared to women who breastfed less than 4 months, a greater percentage of women who breastfed 4 months or longer had private pumping spaces (46.2% vs. 33.0%, *p* = 0.035, not significant after Bonferroni correction) and utilities that supported breastfeeding (85.9% vs. 69.5%, *p* = 0.022, not significant after Bonferroni correction). Among women who breastfed 4 months or longer, 56.3% pumped at work, compared to 13.6% of women who breastfed less than 4 months (*p* = 0.000). Within the work evaluation and experience domain, there were no significant differences by duration of breastfeeding (all *p* > 0.300).

Similar to Table 2, there was a consistent pattern of women with responses of “not sure” to workplace questions in Table 4. Except for other maternity leave, women who breastfed less than 4 months had greater percentages of “not sure” responses than women who breastfed 4 months or longer. Across marginally significant associations for having pumping break times, flexible times for pumping, private pumping spaces, and onsite utilities for pumping, the percentages of women “not sure” were greater for women who breastfed less than 4 months (range: 30.5–49.1%) than women who breastfed 4 months or longer (range: 14.1–32.0%).

### 3.3. Industry

Table 5 presents the maternal demographic, employment, pregnancy, and breastfeeding characteristics stratified by industry (accommodation, healthcare, retail, and other). By industry, the percentage of people aged 18 to 34 was largest and smallest for retail and other industries (84.6% and 73.2%, respectively) and the percentage of people aged 35 and over was largest and smallest for healthcare and accommodation (20.2% and 6.9%, respectively, *p* = 0.023). There was no association between industry and race/ethnicity (*p* = 0.191). Employment status varied significantly by industry with the largest and smallest percent of full-time workers in accommodation and retail (48.3% and 33.8%, respectively), and the largest and smallest percent of part-time workers in retail and healthcare (60.0% and 34.0%, respectively, *p* = 0.017).

Across industries, there were marginally significant differences in how women hoped to feed or if they received information about breastfeeding during pregnancy. The greatest and smallest percentage of women who hoped to only breastfeed were in other industries and accommodation (63.0% and 42.5%, respectively), and the greatest and smallest percentage of women who hoped to only feed with formula were in accommodation and other industries (24.1% and 15.4%, respectively, *p* = 0.029, not significant after Bonferroni correction). The greatest and smallest percentage of women who received information about breastfeeding were in healthcare and accommodation (100.0% and 90.8%, respectively, *p* = 0.017, not significant after Bonferroni correction). Reasons for stopping breastfeeding did not vary significantly by industry (*p* = 0.256).

Table 6 presents the maternal workplace characteristics stratified by industry (accommodation, healthcare, retail, and other). By industry, there were significant and marginally significant differences in policies and culture domain factors. Women working in healthcare and retail had the greatest and smallest percentages of other forms of maternity leave (44.4% and 15.1%, *p* = 0.000). Further, women working in healthcare and accommodation had the greatest and smallest percentages of other forms of maternity leave (20.0% and 3.9%, *p* = 0.003). In addition, there were marginally significant differences in paid maternity leave by industry. The greatest percentages of paid maternity were found among women working in healthcare and other industries and the smallest percentages were found among women working in accommodation and retail (22.1% and 23.3% vs. 7.3% and 15.9%, respectively, *p* = 0.021, not significant after Bonferroni correction).

Within the physical environment and safety climate domain, there were significant and marginally significant differences by industry. Women in healthcare and other industries had the greatest percentages of private pumping spaces in the workplace, compared to accommodation and retail (55.0% and 46.3% vs. 26.0% and 24.2%, respectively, *p* = 0.000). Further, women in healthcare and “other” industries had the greatest percentages of supportive coworkers in the workplace, compared to accommodation and retail (63.9% and 61.1% vs. 42.5% and 42.6%, respectively, *p* = 0.034, not significant after Bonferroni correction), and women in healthcare and “other” industries had the greatest percentages of supportive supervisors in the workplace, compared to accommodation and retail (58.5% and 62.0% vs. 41.6% and 47.5%, respectively, *p* = 0.044, not significant after Bonferroni correction). Within the health behavior domain, greater percentages of women in healthcare and other industries pumped at work, compared to women in accommodation and retail (42.2% and 41.3% vs. 23.7% and 23.7%, respectively, *p* = 0.010). There were no significant differences within the work evaluation and experience domain factors by industry (all *p* > 0.095)

Similar to Table 2 and Table 4, patterns emerged when examining responses of “not sure” in Table 6. Across significant effects, women working in retail had the greatest percentages of “not sure” and women working in other industries had the smallest percentages of ”not sure.”

## 4. Discussion

### 4.1. Findings

Based on previous research and the TWH model, this study explored, among WIC participants and by industry, factors that encourage and/or discourage the initiation and continuation of breastfeeding; pumping at work; and the workplace behaviors, policies, culture, environment, and safety climate associated with breastfeeding and pumping. Our study confirmed the results of a previous study of WIC participants and found that breastfeeding initiation was associated with age, race/ethnicity, and hoping or planning to breastfeed [13]. Further, also similar to this previous study, we found that breastfeeding initiation was not associated with employment status.

Our study did find, however, significant associations between industry and breastfeeding initiation, pumping at work, and the policies and physical environment related to breastfeeding and pumping. Confirming previous findings, we found that women in specific service-oriented industries (i.e., accommodation and retail) reported the lowest rates of breastfeeding initiation and workplace supports for breastfeeding and pumping [12]. Compared to healthcare and all other industries, women who worked in accommodation or retail had the smallest percentages of ever having breastfed, planning to only breastfeed, receiving information on breastfeeding while pregnant, paid maternity leave, a breastfeeding policy, private pumping spaces, and having pumped at work. 

Notably, workplace policy factors supporting breastfeeding (i.e., having paid maternity leave, other maternity leave, and a breastfeeding policy) varied by industry but were not associated with breastfeeding initiation or duration. Further, how a woman hoped to feed and having a private pumping space at work were significantly associated with industry, breastfeeding initiation, and breastfeeding duration. 

Our study confirms previous findings by Snyder et al. (2018) that having a breastfeeding policy, a space to pump, and coworker and supervisor support towards pumping at work vary by industry with the highest rates found among women in a professional industry (i.e., healthcare) [12]. Further, we also found women in service-oriented industries reported the lowest rates of having breastfed for four or more months. In contrast to Snyder et al. (2018), the majority of women in our study did not have or were “not sure” about each of these factors. Further, also unlike Synder et al. (2018), the majority of women in our study had not met their goal when they stopped breastfeeding.

### 4.2. Limitations

This study was cross-sectional and we were unable to assess the temporality of associations such as having received information on breastfeeding, how a woman hoped to feed her child, and subsequent breastfeeding behavior. We were also unable to assess supervisor or coworker support of pumping in the workplace pre-partum and its impact on pumping behavior. Survey responses were based on self-report and subject to several biases including but not limited to response bias, nonresponse bias, recall bias, and observation bias. Women who were employed, initiated breastfeeding, or had difficulty breastfeeding may have been more likely to take the survey, respond to all survey questions, and more accurately remember their breastfeeding experiences.

The survey also directed women who were currently on maternity leave or who had recently left their job to also respond to workplace questions. This may have resulted in increased rates of employment, having breastfed, and difficulties breastfeeding than in the entire WIC population. Due to this design effect, we were also unable to reliably identify and analyze the rates of women who were pregnant or gave birth and decided to no longer work (and their workplace environment).

Income, a key factor in the initiation and duration of breastfeeding, was not collected in this study. However, we assumed study participants were of a similar, lower socioeconomic status given they had qualified for the WIC program. All WIC families are at or below 185% of the family poverty level unless they adjunctively qualified through participation in the Supplemental Nutrition Assistance Program, Temporary Assistance for Needy Families Program, and/or Medicaid. The types of jobs and industries reported in our study reflect the low to moderate income of WIC participants. Our study also did not collect the age of the child and we were unable to determine who was still on paid maternity leave at the time of survey administration. Categories for industry were especially broad due to small sample sizes and we were not able to calculate numerous factors related to industry and occupation, employment, and the workplace including occupation alone, additional industries, size of the workplace (greater than or less than 50 people), and exempt versus non-exempt jobs.

Many breastfeeding studies focusing on workplace support by employment type are limited to their sample population [12,21]. This study is limited to women participating in the WIC program and/or mothers with children participating in the WIC program and our findings may not be generalizable outside of this population. 

## 5. Conclusions

### 5.1. Implications

Our study illustrates how breastfeeding worksite supports are critical to TWH and well-being and suggests that women with a supportive environment are more likely to initiate breastfeeding and breastfeed longer compared to those without a supportive environment. When women begin the WIC program, they are often overwhelmed with the demands of working and caring for their family on a limited income. Although women are encouraged to explore their specific workplace policies and environment during breastfeeding education sessions and counseling appointments, these factors may contribute to the high rates of “not sure” reported in this study. Recently, there has been increased attention to breastfeeding support within the workplace to ensure compliance with the Patient Protection and Affordable Care Act (2010), which amended Section 7 of the Fair Labor Standards Act to require employers to provide: (1) “reasonable break time for an employee to express breast milk for her nursing child for 1 year after the child’s birth each time such employee has need to express the milk,” and (2) “a place, other than a bathroom, that is shielded from view and free from intrusion from coworkers and the public, which may be used by an employee to express breast milk.” [37,38]. This study was conducted six years after the law was enacted and women still reported not having space or flexible time at work to express breast milk. 

### 5.2. Recommendations

Currently, there are numerous ambiguities across breastfeeding research, policies, and practice at the state and federal level. There is a need to make clear connections between federal statutes, public health research, official company-specific policies, and actual practices in the workplace, especially the transparent designation of a workplace contact for breastfeeding policies and practice. Human resource or occupational health personnel in the workforce who write policies or design lactation space should be educated on core components that make up comprehensive lactation programs. Managers and supervisors need to be aware of the unique, acute, and transient needs of breastfeeding mothers returning to work specific to their job class, duties, schedules, and locations. Providing education and training directed toward leadership, supervisors, and human resource personnel regarding the benefits of breastfeeding for both employees and employers and the supports needed when returning to work should be included in any TWH framework. In addition, our study found that many women were unaware of their employer’s benefits for lactation support after returning to work, demonstrating the need for improved communication and awareness about the benefits of health-related employer policies in the context of the TWH framework.

### 5.3. Future Research

Additional studies with larger sample sizes of women participating in WIC, characterizing the barriers to breastfeeding associated with specific industries and subpopulations, would inform future interventions, policy development, and education for employers of this population. Currently, lack of support for breastfeeding across industries is only discussed anecdotally, especially within the WIC program. Given our sample population, we suggest that an equality lens be considered in providing and communicating lactation support; studies should examine whether or not women are afraid to ask about the breastfeeding policies in their workplace, how employers disseminate health benefit information, and the availability and visibility of spaces for breastfeeding in the workplace. More broadly, learning about workplace policies and attitudes towards breastfeeding often involves speaking with coworkers and supervisors suggesting worksite power imbalances between employee and employer be examined. Future studies should also consider the high rates of “not sure” reported in this study in response to questions about workplace support and policies for breastfeeding. In order to better educate WIC participants, methods to improve the provision or dissemination of informational materials should be explored.

### 5.4. Final Remarks

In breastfeeding research, policy, and practice a variety of social justices are being addressed by agencies, leaders, and grass roots groups that believe breastfeeding is a human right that should not be denied or sacrificed when returning to work after having a baby. The priorities for these organizations include equal access to prenatal and postpartum lactation care and support, as well as targeted, evidence-based approaches to address the large disparities in workplace accommodations found in this study. 

## Figures and Tables

**Table 1:** Maternal Demographic, Employment, and Pregnancy Characteristics by Breastfeeding Initiation (Never versus Ever).

Characteristic				Breastfed		*p*-Value ^1^ Sig.^2^
			Overall (*n* = 669)	Never (*n* = 119)	Ever (*n* = 550)	
Demographics	Age (years)	15 to 17	0.6% (*n* = 4)	2.5% (*n* = 3)	0.2% (*n* = 1)	0.025 *
		18 to 34	74.9% (*n* = 501)	78.2% (*n* = 93)	74.2% (*n* = 408)	
		35 and over	12.6% (*n* = 84)	8.4% (*n* = 10)	13.5% (*n* = 74)	
		Not Reported	12.0% (*n* = 80)	10.9% (*n* = 13)	12.2% (*n* = 67)	
	Race/Ethnicity	Other	14.9% (*n* = 100)	9.2% (*n* = 11)	16.2% (*n* = 89)	0.123
		Non-Hispanic White	84.9% (*n* = 568)	90.8% (*n* = 108)	83.6% (*n* = 460)	
		Not Reported	0.1% (*n* = 1)	0.0% (*n* = 0)	0.2% (*n* = 1)	
Employment	Status	Full time	26.9% (*n* = 180)	28.6% (*n* = 34)	26.5% (*n* = 146)	0.723
		Part time	25.6% (*n* = 171)	21.8% (*n* = 26)	26.4% (*n* = 145)	
		Other	47.2% (*n* = 316)	49.6% (*n* = 59)	46.7% (*n* = 257)	
		Not Reported	0.3% (*n* = 2)	0.0% (*n* = 0)	0.4% (*n* = 2)	
	Industry	Accommodation	22.8% (*n* = 84)	33.8% (*n* = 22)	20.4% (*n* = 62)	0.011 *
		Healthcare	25.2% (*n* = 93)	20.0% (*n* = 13)	26.3% (*n* = 80)	
		Retail	17.6% (*n* = 65)	24.6% (*n* = 16)	16.1% (*n* = 49)	
		Other	34.4% (*n* = 127)	21.5% (*n* = 14)	37.2% (*n* = 113)	
During Pregnancy	Hoping to Feed	Combination	29.0% (*n* = 194)	27.7% (*n* = 33)	29.3% (*n* = 161)	0.000 *
		Breastfeeding only	55.0% (*n* = 368)	12.6% (*n* = 15)	64.2% (*n* = 353)	
		Formula only	13.3% (*n* = 89)	56.3% (*n* = 67)	4.0% (*n* = 22)	
		Not Reported	2.7% (*n* = 18)	3.4% (*n* = 4)	2.5% (*n* = 14)	
	Received Information	Yes	95.1% (*n* = 636)	92.4% (*n* = 110)	95.6% (*n* = 526)	0.224
		No	4.0% (*n* = 27)	5.9% (*n* = 7)	3.6% (*n* = 20)	
		Not Reported	0.9% (*n* = 6)	1.7% (*n* = 2)	0.7% (*n* = 4)	

^1^*p*-Value based on Fisher’s exact tests or Fisher’s exact tests with Freeman and Halton’s adaptations for RxC tables. ^2^ Statistical significance (*) was based on an alpha of 0.05 with Bonferroni correction based on the number of comparisons within each category or domain (with no correction for demographic variables). A Monte Carlo estimation with 10,000,000 samples was used to calculate Fisher–Freeman–Halton statistics for tables with industry. Estimates were considered marginally significant (^) if they met the alpha criteria of 0.05 but were no longer significant after Bonferroni correction.

**Table 2:** Maternal Workplace Characteristics by Breastfeeding Initiation (Never versus Ever).

TWH Domain ^1^	Characteristic			Breastfed		*p*-Value ^2^ Sig.^3^
			Overall (*n* = 669)	Never (*n* = 119)	Ever (*n* = 550)	
Policies and Culture	Paid Maternity Leave	Yes	15.8% (*n* = 76)	13.8% (*n* = 12)	16.2% (*n* = 64)	0.764
		No	61.6% (*n* = 297)	60.9% (*n* = 53)	61.8% (*n* = 244)	
		Not Sure	22.6% (*n* = 109)	25.3% (*n* = 22)	22.0% (*n* = 87)	
	Other Maternity Leave	Yes	25.3% (*n* = 97)	23.0% (*n* = 17)	25.9% (*n* = 80)	0.821
		No	34.7% (*n* = 133)	33.8% (*n* = 25)	35.0% (*n* = 108)	
		Not Sure	39.9% (*n* = 153)	43.2% (*n* = 32)	39.2% (*n* = 121)	
	Breastfeeding Policy	Yes	10.9% (*n* = 49)	8.8% (*n* = 7)	11.3% (*n* = 42)	0.498
		No	37.7% (*n* = 170)	33.8% (*n* = 27)	38.5% (*n* = 143)	
		Not Sure	51.4% (*n* = 232)	57.5% (*n* = 46)	50.1% (*n* = 186)	
	Seen Policy	Yes	12.2% (*n* = 42)	8.5% (*n* = 5)	12.9% (*n* = 37)	0.480
		No	61.2% (*n* = 211)	59.3% (*n* = 35)	61.5% (*n* = 176)	
		Not Sure	26.7% (*n* = 92)	32.2% (*n* = 19)	25.5% (*n* = 73)	
	Pumping Break Times	Yes	45.5% (*n* = 206)	25.9% (*n* = 21)	49.7% (*n* = 185)	0.000 *
		No	14.8% (*n* = 67)	17.3% (*n* = 14)	14.2% (*n* = 53)	
		Not Sure	39.7% (*n* = 180)	56.8% (*n* = 46)	36.0% (*n* = 134)	
	Flexible Breaks	Yes	81.1% (*n* = 163)	85.0% (*n* = 17)	80.7% (*n* = 146)	1.000
		No	12.4% (*n* = 25)	10.0% (*n* = 2)	12.7% (*n* = 23)	
		Not Sure	6.5% (*n* = 13)	5.0% (*n* = 1)	6.6% (*n* = 12)	
Physical Environment and Safety Climate	Private Pumping Space	Yes	36.9% (*n* = 162)	19.0% (*n* = 15)	40.8% (*n* = 147)	0.000 *
		No	36.7% (*n* = 161)	39.2% (*n* = 31)	36.1% (*n* = 130)	
		Not Sure	26.4% (*n* = 116)	41.8% (*n* = 33)	23.1% (*n* = 83)	
	Supportive Coworkers	Yes	49.2% (*n* = 215)	36.6% (*n* = 26)	51.6% (*n* = 189)	0.014 *
		No	7.6% (*n* = 33)	4.2% (*n* = 3)	8.2% (*n* = 30)	
		Not Sure	43.2% (*n* = 189)	59.2% (*n* = 42)	40.2% (*n* = 147)	
	Supportive Supervisors	Yes	48.8% (*n* = 211)	38.0% (*n* = 27)	51.0% (*n* = 184)	0.011 *
		No	7.6% (*n* = 33)	2.8% (*n* = 2)	8.6% (*n* = 31)	
		Not Sure	43.5% (*n* = 188)	59.2% (*n* = 42)	40.4% (*n* = 146)	

^1^ For the Total Worker Health well-being domain questions, survey participants were only instructed to reply for their current job, and if they were currently on maternity leave or recently left their job, they were asked to respond for their most recent job. ^2^ *p*-Value was based on Fisher’s exact tests or Fisher’s exact tests with Freeman and Halton’s adaptations for RxC tables. ^3^ Statistical significance (*) based on an alpha of 0.05 with Bonferroni correction based on the number of comparisons within each category or domain (with no correction for demographic variables). A Monte Carlo estimation with 10,000,000 samples was used to calculate Fisher–Freeman–Halton statistics for tables with industry. Estimates were considered marginally significant (^) if they met the alpha criteria of 0.05 but were no longer significant after Bonferroni correction.

**Table 3:** Maternal Demographic, Employment, Pregnancy, and Breastfeeding Characteristics by Duration of Breastfeeding (<4 Months vs. ≥4 Months).

Characteristic			Duration (Months, *n* = 307)		*p*-Value ^1^ Sig.^2^
			<4 (*n* = 139)	≥4 (*n* = 168)	
Demographics	Age (years)	15 to 17	0.7% (*n* = 1)	0.0% (*n* = 0)	0.270
		18 to 34	77.0% (*n* = 107)	72.6% (*n* = 122)	
		35 and over	11.5% (*n* = 16)	17.9% (*n* = 30)	
		Not Reported	10.8% (*n* = 15)	9.5% (*n* = 16)	
	Race/Ethnicity	Other	10.1% (*n* = 14)	16.7% (*n* = 28)	0.099
		Non-Hispanic White	89.9% (*n* = 125)	83.3% (*n* = 140)	
		Not Reported	0.0 % (*n* = 0)	0.0 % (*n* = 0)	
Employment	Status	Full time	25.2% (*n* = 35)	29.2% (*n* = 49)	0.773
		Part time	28.8% (*n* = 40)	28.0% (*n* = 47)	
		Other	46.0% (*n* = 64)	42.3% (*n* = 71)	
		Not Reported	0.0% (*n* = 0)	0.6% (*n* = 1)	
	Industry	Accommodation	23.8% (*n* = 19)	16.0% (*n* = 15)	0.650
		Healthcare	27.5% (*n* = 22)	29.8% (*n* = 28)	
		Retail	16.3% (*n* = 13)	18.1% (*n* = 17)	
		Other	32.5% (*n* = 26)	36.2% (*n* = 34)	
During Pregnancy	Hoping to Feed	Combination	42.4% (*n* = 59)	23.8% (*n* = 40)	0.000 *
		Breastfeeding only	50.4% (*n* = 70)	73.8% (*n* = 124)	
		Formula only	4.3% (*n* = 6)	0.6% (*n* = 1)	
		Not Reported	2.9% (*n* = 4)	1.8% (*n* = 3)	
	Received Information	Yes	96.4% (*n* = 134)	97.6% (*n* = 164)	0.736
		No	3.6% (*n* = 5)	2.4% (*n* = 4)	
		Not Reported	0.0 % (*n* = 0)	0.0 % (*n* = 0)	
Reason for Stopping Breastfeeding	Physiological	Yes	86.6% (*n* = 116)	64.7% (*n* = 99)	0.000 *
		No	13.4% (*n* = 18)	35.3% (*n* = 54)	
	Other Commitments	Yes	23.1% (*n* = 31)	22.2% (*n* = 34)	0.888
		No	76.9% (*n* = 103)	77.8% (*n* = 119)	
	Met Goal	Yes	1.5% (*n* = 2)	28.1% (*n* = 43)	0.000 *
		No	98.5% (*n* = 132)	71.9% (*n* = 110)	

^1^*p*-Value was based on Fisher’s exact tests or Fisher’s exact tests with Freeman and Halton’s adaptations for RxC tables. ^2^ Statistical significance (*) was based on an alpha of 0.05 with Bonferroni correction based on the number of comparisons within each category or domain (with no correction for demographic variables). A Monte Carlo estimation with 10,000,000 samples was used to calculate Fisher–Freeman–Halton statistics for tables with industry. Estimates were considered marginally significant (^) if they met the alpha criteria of 0.05 but were no longer significant after Bonferroni correction.

**Table 4:** Maternal Workplace Characteristics by Duration of Breastfeeding (<4 Months Versus ≥4 Months).

TWH Domain ^1^	Characteristic		Duration (Months, *n* = 307)		*p*-Value ^2^ Sig.^3^
			<4 (*n* = 139)	≥4 (*n* = 168)	
Policies and Culture	Paid Maternity Leave	Yes	17.9% (*n* = 20)	16.2% (*n* = 21)	0.891
		No	55.4% (*n* = 62)	58.5% (*n* = 76)	
		Not Sure	26.8% (*n* = 30)	25.4% (*n* = 33)	
	Other Maternity Leave	Yes	31.9% (*n* = 29)	18.4% (*n* = 18)	0.055
		No	24.2% (*n* = 22)	36.7% (*n* = 36)	
		Not Sure	44.0% (*n* = 40)	44.9% (*n* = 44)	
	Breastfeeding Policy	Yes	8.3% (*n* = 9)	13.3% (*n* = 16)	0.240
		No	29.6% (*n* = 32)	35.0% (*n* = 42)	
		Not Sure	62.0% (*n* = 67)	51.7% (*n* = 62)	
	Seen Policy	Yes	13.0% (*n* = 10)	15.1% (*n* = 14)	0.361
		No	54.5% (*n* = 42)	62.4% (*n* = 58)	
		Not Sure	32.5% (*n* = 25)	22.6% (*n* = 21)	
	Pumping Break Times	Yes	39.8% (*n* = 43)	53.3% (*n* = 65)	0.032
		No	11.1% (*n* = 12)	14.8% (*n* = 18)	
		Not Sure	49.1% (*n* = 53)	32.0% (*n* = 39)	
	Flexible Breaks	Yes	76.2% (*n* = 32)	92.1% (*n* = 58)	0.077
		No	16.7% (*n* = 7)	6.3% (*n* = 4)	
		Not Sure	7.1% (*n* = 3)	1.6% (*n* = 1)	
Physical Environment and Safety Climate	Private Pumping Space	Yes	33.0% (*n* = 34)	46.2% (*n* = 54)	0.035
		No	33.0% (*n* = 34)	34.2% (*n* = 40)	
		Not Sure	34.0% (*n* = 35)	19.7% (*n* = 23)	
	Onsite Items	Utilities, Yes	69.5% (*n* = 41)	85.9% (*n* = 73)	0.022
		Utilities, No	30.5% (*n* = 18)	14.1% (*n* = 12)	
		Physical, Yes	96.6% (*n* = 57)	89.4% (*n* = 76)	0.200
		Physical, No	3.4% (*n* = 2)	10.6% (*n* = 9)	
	Supportive Coworkers	Yes	46.6% (*n* = 48)	56.2% (*n* = 68)	0.289
		No	6.8% (*n* = 7)	7.4% (*n* = 9)	
		Not Sure	46.6% (*n* = 48)	36.4% (*n* = 44)	
	Supportive Supervisors	Yes	46.1% (*n* = 47)	58.7% (*n* = 71)	0.155
		No	6.9% (*n* = 7)	6.6% (*n* = 8)	
		Not Sure	47.1% (*n* = 48)	34.7% (*n* = 42)	
Health Behavior	Pumped at Work	Yes	13.6% (*n* = 14)	56.3% (*n* = 67)	0.000
		No	86.4% (*n* = 89)	43.7% (*n* = 52)	
Work Evaluation and Experience	Reaction to Pumping	Any negative	35.7% (*n* = 5)	29.2% (*n* = 19)	0.750
		Positive/None	64.3% (*n* = 9)	70.8% (*n* = 46)	
	Pumped Longer if Easier	Yes	31.6% (*n* = 31)	36.5% (*n* = 42)	0.513
		No	29.6% (*n* = 29)	32.2% (*n* = 37)	
		Not Sure	38.8% (*n* = 38)	31.3% (*n* = 36)	
	Factors	Policy/Culture, Yes	72.4% (*n* = 21)	61.9% (*n* = 26)	0.447
		Policy/Culture, No	27.6% (*n* = 8)	38.1% (*n* = 16)	
		Environment, Yes	62.1% (*n* = 18)	47.6% (*n* = 20)	0.333
		Environment, No	37.9% (*n* = 11)	52.4% (*n* = 22)	

^1^ For the Total Worker Health well-being domain questions, survey participants were only instructed to reply for their current job, and if they were currently on maternity leave or recently left their job, they were asked to respond for their most recent job. ^2^ *p*-Value was based on Fisher’s exact tests or Fisher’s exact tests with Freeman and Halton’s adaptations for RxC tables. ^3^ Statistical significance (*) was based on an alpha of 0.05 with Bonferroni correction based on the number of comparisons within each category or domain (with no correction for demographic variables). A Monte Carlo estimation with 10,000,000 samples was used to calculate Fisher–Freeman–Halton statistics for tables with industry. Estimates were considered marginally significant (^) if they met the alpha criteria of 0.05 but were no longer significant after Bonferroni correction.

**Table 5:** Maternal Demographic, Employment, Pregnancy, and Breastfeeding Characteristics by Industry.

Characteristic			Industry (*n* = 373)				*p*-Value ^1^ Sig.^2^
			Accommodation	Healthcare	Retail	Other	
			(*n* = 87)	(*n* = 94)	(*n* = 65)	(*n* = 127)	
Demographics	Age (years)	15 to 17	0.0% (*n* = 0)	0.0% (*n* = 0)	0.0% (*n* = 0)	0.0% (*n* = 0)	0.023 *
		18 to 34	77.0% (*n* = 67)	74.5% (*n* = 70)	84.6% (*n* = 55)	73.2% (*n* = 93)	
		35 and over	6.9% (*n* = 6)	20.2% (*n* = 19)	9.2% (*n* = 6)	17.3% (*n* = 22)	
		Not Reported	16.1% (*n* = 14)	5.3% (*n* = 5)	6.2% (*n* = 4)	9.4% (*n* = 12)	
	Race/Ethnicity	Other	17.2% (*n* = 15)	10.6% (*n* = 10)	6.2% (*n* = 4)	14.2% (*n* = 18)	0.191
		Non-Hispanic White	82.8% (*n* = 72)	88.3% (*n* = 83)	93.8% (*n* = 61)	85.8% (*n* = 109)	
		Not Reported	0.0% (*n* = 0)	1.1% (*n* = 1)	0.0% (*n* = 0)	0.0% (*n* = 0)	
Employment	Status	Full time	48.3% (*n* = 42)	46.8% (*n* = 44)	33.8% (*n* = 22)	39.4% (*n* = 50)	0.017 *
		Part time	41.4% (*n* = 36)	34.0% (*n* = 32)	60.0% (*n* = 39)	42.5% (*n* = 54)	
		Other	10.3% (*n* = 9)	19.1% (*n* = 18)	6.2% (*n* = 4)	18.1% (*n* = 23)	
		Not Reported	0.0% (*n* = 0)	0.0% (*n* = 0)	0.0% (*n* = 0)	0.0% (*n* = 0)	
During Pregnancy	Hoping to Feed	Combination	31.0% (*n* = 27)	34.0% (*n* = 32)	30.8% (*n* = 20)	26.8% (*n* = 34)	0.029 ^
		Breastfeeding only	42.5% (*n* = 37)	53.2% (*n* = 50)	49.2% (*n* = 32)	63.0% (*n* = 80)	
		Formula only	24.1% (*n* = 21)	9.6% (*n* = 9)	15.4% (*n* = 10)	7.1% (*n* = 9)	
		Not Reported	2.3% (*n* = 2)	3.2% (*n* = 3)	4.6% (*n* = 3)	3.1% (*n* = 4)	
	Received Information	Yes	90.8% (*n* = 79)	100.0% (*n* = 94)	96.9% (*n* = 63)	94.5% (*n* = 120)	0.017 *
		No	9.2% (*n* = 8)	0.0% (*n* = 0)	3.1% (*n* = 2)	4.7% (*n* = 6)	
		Not Reported	0.0% (*n* = 0)	0.0% (*n* = 0)	0.0% (*n* = 0)	0.8% (*n* = 1)	
Reason for Stopping Breastfeeding	Physiological	Yes	63.9% (*n* = 39)	73.9% (*n* = 51)	58.3% (*n* = 28)	60.7% (*n* = 54)	0.249
		No	36.1% (*n* = 22)	26.1% (*n* = 18)	41.7% (*n* = 20)	39.3% (*n* = 35)	
	Other Commitments	Yes	26.2% (*n* = 16)	14.5% (*n* = 10)	12.5% (*n* = 6)	28.1% (*n* = 25)	0.059
		No	73.8% (*n* = 45)	85.5% (*n* = 59)	87.5% (*n* = 42)	71.9% (*n* = 64)	
	Met Goal	Yes	6.6% (*n* = 4)	10.1% (*n* = 7)	14.6% (*n* = 7)	16.9% (*n* = 15)	0.256
		No	93.4% (*n* = 57)	89.9% (*n* = 62)	85.4% (*n* = 41)	83.1% (*n* = 74)	

^1^*p*-Value was based on Fisher’s exact tests or Fisher’s exact tests with Freeman and Halton’s adaptations for RxC tables. ^2^ Statistical significance (*) was based on an alpha of 0.05 with Bonferroni correction based on the number of comparisons within each category or domain (with no correction for demographic variables). A Monte Carlo estimation with 10,000,000 samples was used to calculate Fisher–Freeman–Halton statistics for tables with industry. Estimates were considered marginally significant (^) if they met the alpha criteria of 0.05 but were no longer significant after Bonferroni correction.

**Table 6:** Maternal Workplace Characteristics by Industry.

TWH Domain ^1^	Characteristic	Industry (*n* = 373)					*p*-Value ^2^ Sig. ^3^
		Accommodation	Health care	Retail		Other	
		(*n* = 87)	(*n* = 94)	(*n* = 65)		(*n* = 127)	
Policies and Culture	Paid Maternity Leave	Yes	7.3% (*n* = 6)	22.1% (*n* = 19)	15.9% (*n* = 10)	23.3% (*n* = 27)	0.021 ^
		No	79.3% (*n* = 65)	58.1% (*n* = 50)	61.9% (*n* = 39)	62.9% (*n* = 73)	
		Not Sure	13.4% (*n* = 11)	19.8% (*n* = 17)	22.2% (*n* = 14)	13.8% (*n* = 16)	
	Other Maternity Leave	Yes	16.4% (*n* = 12)	44.4% (*n* = 28)	15.1% (*n* = 8)	25.9% (*n* = 22)	0.000 *
		No	53.4% (*n* = 39)	17.5% (*n* = 11)	35.8% (*n* = 19)	47.1% (*n* = 40)	
		Not Sure	30.1% (*n* = 22)	38.1% (*n* = 24)	49.1% (*n* = 26)	27.1% (*n* = 23)	
	Breastfeeding Policy	Yes	3.9% (*n* = 3)	20.0% (*n* = 17)	8.5% (*n* = 5)	17.0% (*n* = 19)	0.003 *
		No	49.4% (*n* = 38)	36.5% (*n* = 31)	28.8% (*n* = 17)	43.8% (*n* = 49)	
		Not Sure	46.8% (*n* = 36)	43.5% (*n* = 37)	62.7% (*n* = 37)	39.3% (*n* = 44)	
	Seen Policy	Yes	3.3% (*n* = 2)	18.2% (*n* = 12)	11.4% (*n* = 5)	19.8% (*n* = 16)	0.096
		No	73.8% (*n* = 45)	65.2% (*n* = 43)	68.2% (*n* = 30)	61.7% (*n* = 50)	
		Not Sure	23.0% (*n* = 14)	16.7% (*n* = 11)	20.5% (*n* = 9)	18.5% (*n* = 15)	
	Pumping Break Times	Yes	35.8% (*n* = 29)	59.0% (*n* = 49)	44.4% (*n* = 28)	54.0% (*n* = 61)	0.071
		No	18.5% (*n* = 15)	8.4% (*n* = 7)	14.3% (*n* = 9)	13.3% (*n* = 15)	
		Not Sure	45.7% (*n* = 37)	32.5% (*n* = 27)	41.3% (*n* = 26)	32.7% (*n* = 37)	
	Flexible Breaks	Yes	72.4% (*n* = 21)	83.3% (*n* = 40)	85.2% (*n* = 23)	84.7% (*n* = 50)	0.788
		No	17.2% (*n* = 5)	12.5% (*n* = 6)	11.1% (*n* = 3)	8.5% (*n* = 5)	
		Not Sure	10.3% (*n* = 3)	4.2% (*n* = 2)	3.7% (*n* = 1)	6.8% (*n* = 4)	
Physical Environment and Safety Climate	Private Pumping Space	Yes	26.0% (*n* = 20)	55.0% (*n* = 44)	24.2% (*n* = 15)	46.3% (*n* = 50)	0.000 *
		No	54.5% (*n* = 42)	21.3% (*n* = 17)	50.0% (*n* = 31)	34.3% (*n* = 37)	
		Not Sure	19.5% (*n* = 15)	23.8% (*n* = 19)	25.8% (*n* = 16)	19.4% (*n* = 21)	
	Onsite Items	Utilities, Yes	76.1% (*n* = 35)	79.4% (*n* = 50)	79.3% (*n* = 23)	83.3% (*n* = 60)	0.797
		Utilities, No	23.9% (*n* = 11)	20.6% (*n* = 13)	20.7% (*n* = 6)	16.7% (*n* = 12)	
		Physical, Yes	91.3% (*n* = 42)	93.7% (*n* = 59)	86.2% (*n* = 25)	93.1% (*n* = 67)	0.602
		Physical, No	8.7% (*n* = 4)	6.3% (*n* = 4)	13.8% (*n* = 4)	6.9% (*n* = 5)	
	Supportive Coworkers	Yes	42.5% (*n* = 34)	63.9% (*n* = 53)	42.6% (*n* = 26)	61.1% (*n* = 66)	0.034 ^
		No	8.8% (*n* = 7)	4.8% (*n* = 4)	6.6% (*n* = 4)	6.5% (*n* = 7)	
		Not Sure	48.8% (*n* = 39)	31.3% (*n* = 26)	50.8% (*n* = 31)	32.4% (*n* = 35)	
	Supportive Supervisors	Yes	41.6% (*n* = 32)	58.5% (*n* = 48)	47.5% (*n* = 29)	62.0% (*n* = 67)	0.044 ^
		No	14.3% (*n* = 11)	3.7% (*n* = 3)	8.2% (*n* = 5)	5.6% (*n* = 6)	
		Not Sure	44.2% (*n* = 34)	37.8% (*n* = 31)	44.3% (*n* = 27)	32.4% (*n* = 35)	
Health Behavior	Pumped at Work	Yes	23.7% (*n* = 18)	42.2% (*n* = 35)	23.7% (*n* = 14)	41.3% (*n* = 45)	0.010 *
		No	76.3% (*n* = 58)	57.8% (*n* = 48)	76.3% (*n* = 45)	58.7% (*n* = 64)	
Work Evaluation and Experience	Reaction to Pumping	Any negative	55.6% (*n* = 10)	34.3% (*n* = 12)	53.8% (*n* = 7)	26.7% (*n* = 12)	0.095
		Positive / None	44.4% (*n* = 8)	65.7% (*n* = 23)	46.2% (*n* = 6)	73.3% (*n* = 33)	
	Pumped Longer if Easier	Yes	35.8% (*n* = 24)	38.6% (*n* = 27)	20.4% (*n* = 10)	36.6% (*n* = 34)	0.250
		No	28.4% (*n* = 19)	30.0% (*n* = 21)	30.6% (*n* = 15)	34.4% (*n* = 32)	
		Not Sure	35.8% (*n* = 24)	31.4% (*n* = 22)	49.0% (*n* = 24)	29.0% (*n* = 27)	
	Factors	Policy/Culture, Yes	69.6% (*n* = 16)	53.8% (*n* = 14)	60.0% (*n* = 6)	69.7% (*n* = 23)	0.554
		Policy/Culture, No	30.4% (*n* = 7)	46.2% (*n* = 12)	40.0% (*n* = 4)	30.3% (*n* = 10)	
		Environment, Yes	60.9% (*n* = 14)	34.6% (*n* = 9)	50.0% (*n* = 5)	48.5% (*n* = 16)	0.335
		Environment, No	39.1% (*n* = 9)	65.4% (*n* = 17)	50.0% (*n* = 5)	51.5% (*n* = 17)	

^1^ For the Total Worker Health well-being domain questions, survey participants were only instructed to reply for their current job, and if they were currently on maternity leave or recently left their job, they were asked to respond for their most recent job. ^2^ *p*-Value was based on Fisher’s exact tests or Fisher’s exact tests with Freeman and Halton’s adaptations for RxC tables. ^3^ Statistical significance (*) was based on an alpha of 0.05 with Bonferroni correction based on the number of comparisons within each category or domain (with no correction for demographic variables). A Monte Carlo estimation with 10,000,000 samples was used to calculate Fisher–Freeman–Halton statistics for tables with industry. Estimates were considered marginally significant (^) if they met the alpha criteria of 0.05 but were no longer significant after Bonferroni correction.
